# Determining social and population structures requires multiple approaches: A case study of the desert ant *Cataglyphis israelensis*

**DOI:** 10.1002/ece3.4535

**Published:** 2018-12-10

**Authors:** Tali Reiner Brodetzki, Abraham Hefetz

**Affiliations:** ^1^ School of Zoology, George S. Wise Faculty of Life Sciences Tel Aviv University Tel Aviv Israel

**Keywords:** cuticular hydrocarbons, microsatellite analysis, polydomy

## Abstract

The remarkable diversity of ant social organization is reflected in both their life history and population kin structure. Different species demonstrate a high variation with respect to both social structure and mating strategies: from the ancestral colony type that is composed of a single queen (monogyny), singly inseminated (monoandry), to the more derived states of colonies headed by a multiply inseminated queen (polyandry), to colonies composed of multiple queens (polygyny) that are either singly or multiply inseminated. Moreover, the population structure of an ant species can range from multicoloniality to polydomy to supercoloniality, and *Cataglyphis* is considered to be a model genus in regard to such diversity. The present study sought to determine the social and population structure of the recently described *C. israelensis* species in Israel. For this purpose we employed a multidisciplinary approach, rather than the commonly used single approach that is mostly based on genetics. Our study encompassed behavior (nest insularity/openness), chemistry (composition of nestmate recognition signals and cuticular hydrocarbons), and genetics (microsatellite polymorphism). Each approach has been shown to possess both advantages and disadvantages, depending on the studied species. Our findings reveal that *C. israelensis *colonies are headed by a single, multiply inseminated queen and that the population structure is polydomous, with each colony comprising one main nest and several additional satellite nests. Moreover, our findings demonstrate that none of the above‐noted approaches, when employed individually, is suitable or sufficient in itself for delineating population structure, thus emphasizing the importance of using multiple approaches when assessing such complex systems.

## 1 INTRODUCTION

Understanding how animal societies are organized constitutes a major question in evolutionary biology (Wilson, 2012).Comparing social and population structures can shed light on the evolution of sociality as well as on other traits (Kennedy et al., 2017). In social Hymenoptera, although social structures and reproductive strategies can vary greatly among species (Boomsma, 2009; Crozier & Pamilo, 1996), they all share the basic colony structure that is presumably driven by kin selection, reinforced by the haplo‐diploid sex determination system, in which within‐colony relatedness is higher than that between colonies, and individuals benefit from inclusive fitness rather than individual fitness (Bourke & Franks, 1995; Hamilton, 1964, 1972 ; Hölldobler & Wilson, 1990; Queller, 1992). However, when there is limited dispersal or variation in the basic colony form, such as multiply mated queens (polyandry) or multiple queens (polygyny), these assumptions do not always hold true. In such cases, within‐colony relatedness is very low (Keller, 1993), and workers from two distinct but neighboring colonies can be more related to each other than to their respective nestmates (Heinze, 2008; Procter et al., 2016; Wright, 1943). It is thus insufficient to assess colony boundaries using genetic methods alone. Another colony‐specific “marker” is that of the cuticular hydrocarbon profile (CHC), which in many ant species is responsible for nestmate recognition. CHC composition is attributed both to genetic and nutritional differences (Liang & Silverman, 2000; Vonshak, Dayan, Foucaud, Estoup, & Hefetz, 2009), and nestmates are assumed to possess similar CHC profiles due to the so‐called “colony gestalt” (Hefetz, 2007; Soroker, Vienne, Hefetz, & Nowbahari, 1994). However, there may be cases in which these profiles could also overlap with individual profiles of workers from other nests, especially if dispersal is limited. Thus, employing additional methods is important when seeking to understand the colony boundaries. One such method is that of behavioral assays: that is, acceptance/rejection of non/nestmates. Such assays, however, need to be designed according to the aggressiveness level of the species. In species that are highly territorial and aggressive, simple dyadic encounters between nestmates and nonnestmates will result in an unequivocal aggressive response, while in less aggressive species similar assays may result in mild responses, irrespective of whether the encountering ants are nestmates or alien (Buczkowski, Kumar, Suib, & Silverman, 2005). In the latter case, assays that determine whether two nests merge into a single colony may provide more unequivocal results (Boulay, Katzav‐Gozansky, Vander Meer, & Hefetz, 2003). To date, only a few studies have employed multiple methods to assess social and population structures (Ellis, Procter, Buckham‐Bonnett, & Robinson, 2017). In the present study, we employed all three methods: genetic (microsatellite analysis), chemical (CHC nest profiles), and behavioral (colony insularity/openness tests), and demonstrate that none of the methods when applied individually is sufficient in itself for delineating colony boundaries.

The genus *Cataglyphis* is considered a good model for social evolution (Boulay et al., 2017; Lenoir, Aron, Cerda, & Hefetz, 2009). It comprises over a hundred species (Agosti, 1990), displaying various social and population structures, ranging from the basic monogynous multicolonial population to polygynous supercolonies (Jowers et al., 2013; Leniaud, Heftez, Grumiau, & Aron, 2011; Timmermans, Grumiau, Hefetz, & Aron, 2010; Timmermans, Hefetz, Fournier, & Aron, 2008). The genus also expresses various reproductive strategies, from sexual to the asexual reproduction of new gynes (Darras, Leniaud, & Aron, 2014; Eyer, Leniaud, Darras, & Aron, 2013; Leniaud, Darras, Boulay, & Aron, 2012). *Cataglyphis* is distributed throughout the Old World deserts and arid habitats. A recently identified new species, *C. israelensis*, is distributed mainly in the north and north‐eastern parts of Israel (Eyer, Seltzer, Reiner‐Brodetzki, & Hefetz, 2017; Ionescu & Eyer, 2016), and is the subject of our study. The study was conducted at the distribution edge of this species along the northern coastline of Israel, presenting a similar ecological niche to that of other recently studied congener species (Leniaud et al., 2011; Saar, Leniaud, Aron, & Hefetz, 2014) that display various forms of sociality.

## 2 METHODS

### 2.1 Sample collection

Desert ants in the genus *Cataglyphis* are diurnal, highly thermophilic ants that are wide spread in arid habitats of the Old World. Their nest demography and architecture seem to be constrained by ecological factors. For example, in habitats along the Mediterranean Sea shores, nest depths are limited compared to the much deeper nests located in inland areas (Clémencet & Doums, 2007). *Cataglyphis* are central‐place foragers, usually scavengers, but also contribute to pollination and seed dispersal (Boulay, Carro, Soriguer, & Cerdá, 2007; Cerda, Retana, & Cros, 1997). The ants forage individually and do not use any form of recruitment other than performing simple invitation behavior when a food source is discovered (Amor, Ortega, Cerdá, & Boulay, 2010). They are also well known for their high navigational ability, using both path integration and landmark guidance mechanisms (Wehner, 2008). Like many other ant species, they commonly have well‐insulated nests and use CHCs as nestmate recognition cues (Hefetz, 2007; Soroker et al., 1994). They present highly diverse reproduction strategies, social structures, and population structures (Boulay et al., 2017; Lenoir et al., 2009).

**Figure 1:**
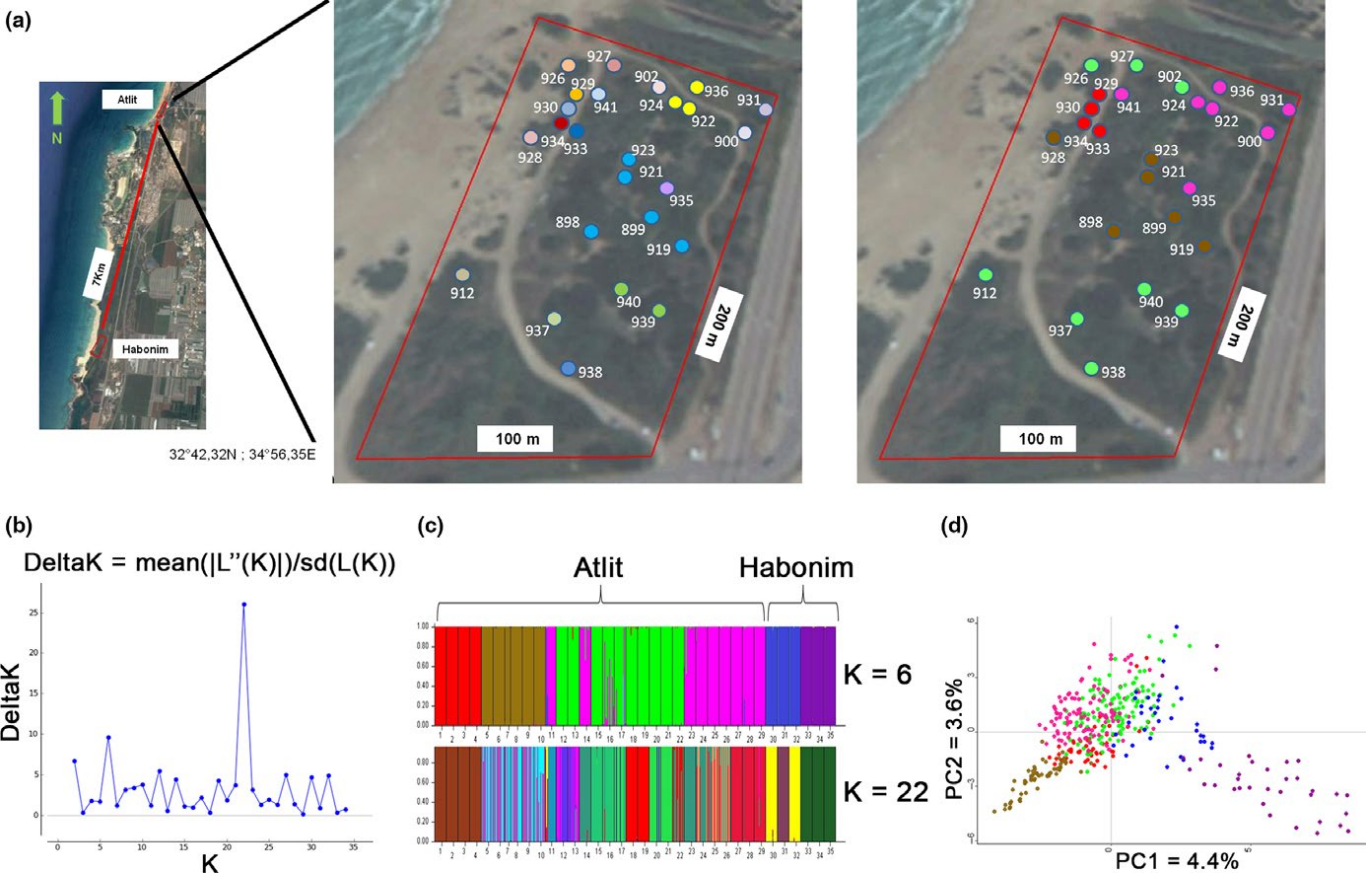
(a) From left to right: study site locations at Atlit and Habonim (7 km apart), and nests sampled at the Atlit site: colors of the nests are according to COLONY matrilines (same colors indicate the same colony as in table ST2), colors of the nests are according to STRUCTUE analysis. (b) STRUCTURE harvester results indicating most probable K number. (c) STRUCTURE results indicating *K* = 6 or 22; individuals are grouped by nest origin, and each assumed colony is color coded. (d) PCA of microsatellite data with colony colors as indicated by STRUCTURE

Nests of *C. israelensis* were collected from two distinct populations on the coastal plain of northern Israel, in semi‐stabilized sand dunes, just south of Mount Carmel: from Atlit (N33 04.607 E35 06.554) comprising 37 nests in a plot of 100 × 200 m; and from Habonim (N32 38.920 E34 55.669; about 7 km south of Atlit; Figure 1a) comprising eight nests in a plot of 100x350 m. Nests were thoroughly excavated to include the queen and all workers, and brood. To ensure that the entire nest was collected, excavation was further extended by an extra 0.5–1 m in all directions after having observed the last gallery. For genetic and chemical analysis, all the nests in the experimental plot were excavated over four consecutive days.

Only ten of these nests contained queens, always a single queen per nest. Because the Habonim population is sparse, and sample size was low for this population, it is included in only some of the analyses. Number of nests in each analysis is provided in Supporting Information Table S1.

### 2.2 Genetic analysis

Since the study area hosts at least two sympatric *Cataglyphis* species in the bicolor group, we first confirmed that all the nests collected indeed belong to *C. israelensis* by genotyping Cytochrom B using one worker per nest (Eyer et al., 2017). CytB primers used: CB1 (Forward) TATGTACTACCATGAGGACAAATATC and CB2 (Reverse) ATTACACCTCCTAATTTATTAGGAAT, annealing temperature was 48°C (Simon et al., 1994). PCR products were purified with the spin‐column PCR purification kit (TIANGEN Biotech), followed by sequencing with the ABI BigDye Terminator v.3.1 Cycle Sequencing Kit (Applied Biosystems, Foster City, CA, USA). Sequencing was performed on an ABI 3,730 Genetic Analyzer (Applied Biosystems). Base calling and sequence reconciliation were inspected by eye and performed using CodonCode Aligner (CodonCode Corporation, Dedham, MA, USA). Sequences were aligned using MUSCLE algorithm (Edgar, 2004) implemented in CodonCode Aligner, together with sequences previously used in Eyer 2017. All samples that were used in this project had the same CytB mitotype, similar to the *C. israelensis* mitotype from the main geographic distribution of the species.

Genetic (microsatellites) analysis was performed on 348 workers from the Atlit population (12*29 nests) and 72 workers (12*6 nests) from the Habonim population. DNA was extracted with 5% CHELEX (BIO‐RAD) and amplified with eight microsatellite markers that had been designed previously for *C. hispanica* (Ch23, Ch08, Ch01, Ch10, Ch11 (Darras, et al., 2014)), *C. cursor* (Cc99, Cc54 (Pearcy, Clémencet, Chameron, Aron, & Doums, 2004), and *C. niger* (Cn02 (Saar et al., 2014) using Type‐it PCR mix (QIAGEN). PCR products were sequenced by ABI3500 sequencer, and genotypes were then visualized with GeneMarker. Microsatellite data were checked for linkage disequlibrium, fixation index, and G‐test using GENEPOP (Raymond & Rousset, 1995), heterozygosity was checked using both GENEPOP and FSTAT (Goudet, 1995), and *F*_st_ was checked using FSTAT. To assess the number of queens, queens’ genotypes, and the number of matings we used the program COLONY (Jones & Wang, 2010). The program STRUCTURE (Pritchard, Stephens, & Donnelly, 2000) was used to evaluate the number of colonies in the population. The number of *K* (number of possible colonies, from 1 to n size) was evaluated by the Evanno method using STRUCTURE Harvester (Earl, 2012). PCA of microsatellite data was performed as described by Ryan *et al.* (2017; using R version 3.3.3; library *adegenet* and *ggplot2* for visualization), and relatedness was estimated using Coancestry (Wang, 2011; Queller GT method).

### 2.3 Cuticular hydrocarbons analysis

For cuticular hydrocarbon analysis, we immersed individual heads in hexane (5–10 ants from each of 36 nests), containing 100 ng/µl of C20 as internal standard. Initial analysis was conducted by gas chromatography/mass spectrometry (GC/MS), using a VF‐5 ms capillary column, temperature‐programed from 60 to 300°C (with 1 min initial hold) at a rate of 10°C per min, with a final hold of 15 min. Compound identification was achieved according to their fragmentation pattern, and the respective retention indices were compared to authentic compounds. Quantitative analyses were performed by flame ionization gas chromatography (GC/FID), using the above running conditions. Peak integration was performed using the program Galaxie Varian 1.9. Only hydrocarbons (a total of 24 compounds), which presumably play a major role in nestmate recognition (Lahav, Soroker, Hefetz, & Vander Meer, 1999), were used in the analyses. The relative amounts of each peak were calculated as a percentage of all the compounds in the analysis. Hydrocarbon profiles of individuals were first plotted using principal component analysis (PCA; prcomp function of *stats* plotting was done by autoplot in *ggplot2*); however, this unsupervised analysis does not provide much information when the differences between samples are small. We then used *k*‐means clustering in order to understand the structuring of the population, and assessed the most probable *K* using *k*‐means function in *stats, *as well as mclust in *mclust* and nbclust in *NbClust* libraries in R. Plot was done by clusplot function in *cluster* library. Hydrocarbon profile specificity was also determined using multivariate statistics, that is, discriminant analysis using the stepwise forward mode, using IBM SPSS statistics 21. This module of the program works initially as a principle component analysis for reducing the number of variables to fit the number of cases and then performs the discriminant function. We used the colonies suggested by the STRUCTURE program for discrimination.

### 2.4 Behavioral assays

For the nest merging experiments, we composed seven queenright (QR) colonies comprising approximately 200 workers that were maintained in nesting boxes prior to the experiment. Using various combinations, we conducted merging assays between two colonies, which were chosen either according to the dissimilarity of CHC profiles or to nest metric distance (Table 1). We also performed merging assays between QR colonies and their neighboring QL nests, with each of the three QL nests being introduced to all of the QR nests, consecutively. Workers of each colony were marked with different colors using nail polish. At the onset of the merging experiment, the two nests were connected to a common foraging arena (size 20 × 20 cm). Sand and food were placed in the foraging arena, while water and cotton balls were placed in the enclosed nests. Scan observations were performed while registering interactions between nonnestmates, including aggression, trophallaxis, adult carrying, and antennation. Observations for the first four hours were carried out for 10 min at 10 min intervals, after which the nests were inspected every 12 hr for up to a week (Boulay et al., 2003). Merging tests between QR and QL nests were performed for the duration of only two hours for each test, with an interval of one day between each test involving the different QR nests.

**Table 1:** List of nests used in the various merging experiments. Behavior occurrence is indicated by +or—(if the behavior did not occur). QR‐queenright nest, QL‐queenless nest

Nest A	Nest B	Merging	Between‐colony trophallaxis	Between‐colony aggression	Maximal distance between colonies (m)
		(+/−)	(+/−)	(+/−)	
7 QR	21 QR	−	−	−	100
8 QR	26 QR	−	−	−	90
101 QR	100 QR	−	−	+	15
100 QR	102 QR	−	−	+	14
101 QR	102 QR	−	−	+	8
100 QR	103/104/105 QL	−	−	+	18,24, and 16
101 QR	103/104/105 QL	−	−	+	7,11, and 3
102 QR	103/104/105 QL	+	+	−	2,11, and 5

### 2.5 Association between genetic, chemical, and geographic distances

The pattern of nest distribution was assessed using the mean distance between nearest neighbors equation as described in Clark and Evans (1954). Pearson correlation was carried out between all types of distances (library *SYNCSA*, library *matrix *for data preparation, library *ggplot2* and *ggpmisc* for visualization in R version 3.3.3). Mantel test was calculated by using the mantel.rtest function (library *ada* in R version 3.3.3) between all types of distances.

To test the hypothesis that the population is polydomous, we performed, using library *dendextend*, cluster analyses using each of the following distances: geographical (calculated from GPS locations), genetical (*F*_st_'s) and chemical (Mahalanobis distances). We then checked for association between the methods, in order to understand how they help to elucidate the population structure, by using cluster analysis (method: average), using the *hclust* algorithm in R version 3.3.3. Since each method resulted in a specific and slightly different dendogram, we assessed their validity by calculating the entanglement level between pairs of dendograms. Entanglement, a function used in the library *dendextend,* is measured by assigning values of consecutive numbers to the left dendogram's labels and then matching these numbers to those assigned to the right dendogram. Entanglement is the *L*‐norm distance between these two vectors. In other words, we take the sum of the absolute difference (each one in the power of *L*; for example, sum(abs(*x*‐*y*)*^L^*) and divide it by the “worst case” entanglement level (e.g., when the right dendogram is the complete reverse of the left dendogram). L determines the penalty level (positive number, usually between 0–2). *L* > 1 means that we give a big penalty for sharp angles; while *L*‐> 0 means that whenever something is not a straight horizontal line, it receives a large penalty If *L* = 0.1, it means that we give higher preference to horizontal lines over nonhorizontal lines. We used *L* = 1.5 to allow for some flexibility; using lower *L* values results in higher entanglement value. A lower entanglement coefficient corresponds to a better alignment.

## 3 RESULTS

### 3.1 Genetic analyses

None of the eight microsatellite markers used in this study showed indications of null alleles or linkage disequilibrium. The number of alleles ranged from 4 to 29. Mean heterozygosity was *H*_o_ = 0.65 (range 0.44–0.69) *H*_e_ = 0.107 (range 0.065–0.128). The fixation index *Fit *was slightly higher than zero (0.001 ± 0.03 *SE* jackknife) indicating random mating in the tested population.* F*_st_ was very low at 0.15, while the within‐nest relatedness was 0.3 (±0.019 *SE* jackknife), which is considerably lower than assumed for a monogynous, monandrous nest (0.75). Nest excavations, in completion, revealed a single queen in nine nests. In 11 nests, COLONY genetic data analyses showed that all of the workers genotypes matched to a single queen, confirming unequivocally monogyny. In the other nests, some of the workers’ genotypes (average of 2.79 workers per nest; *SE* = 0.26) did not perfectly match the assigned queens’ genotype. These workers were observed both in QL (queenless) and in QR (queenright) nests; noteworthy, these QR nests had a single queen. Similar reconstructed matrilines are assumed to belong to the same colony (Supporting Information Table S2 and in Figure 1a). COLONY analysis also indicated polyandry, with an average of five males contributing to worker production (range 3–8; *SE* = 0.28).

Population analysis using the program STRUCTURE indicated that the population consisted in six or 22 colonies out of the 35 nests analyzed (Figure 1a–c). In Habonim the six nests sampled constituted two or three colonies, while in Atlit the 29 nests sampled constituted of four or 20 colonies. The latter value (20 colonies in Atlit) seems less probable in light of the nonuniform genotype distribution per nest, and the fact that the STRUCTURE program assigned different colonies for individuals that had been collected from the same nest. Thus, using this method alone is problematic in such cases. Similar results were produced using PCA (Figure 1d), which enables observation of the similarity of the genotypes between the groups.

### 3.2 Chemical analyses

**Figure 2:**
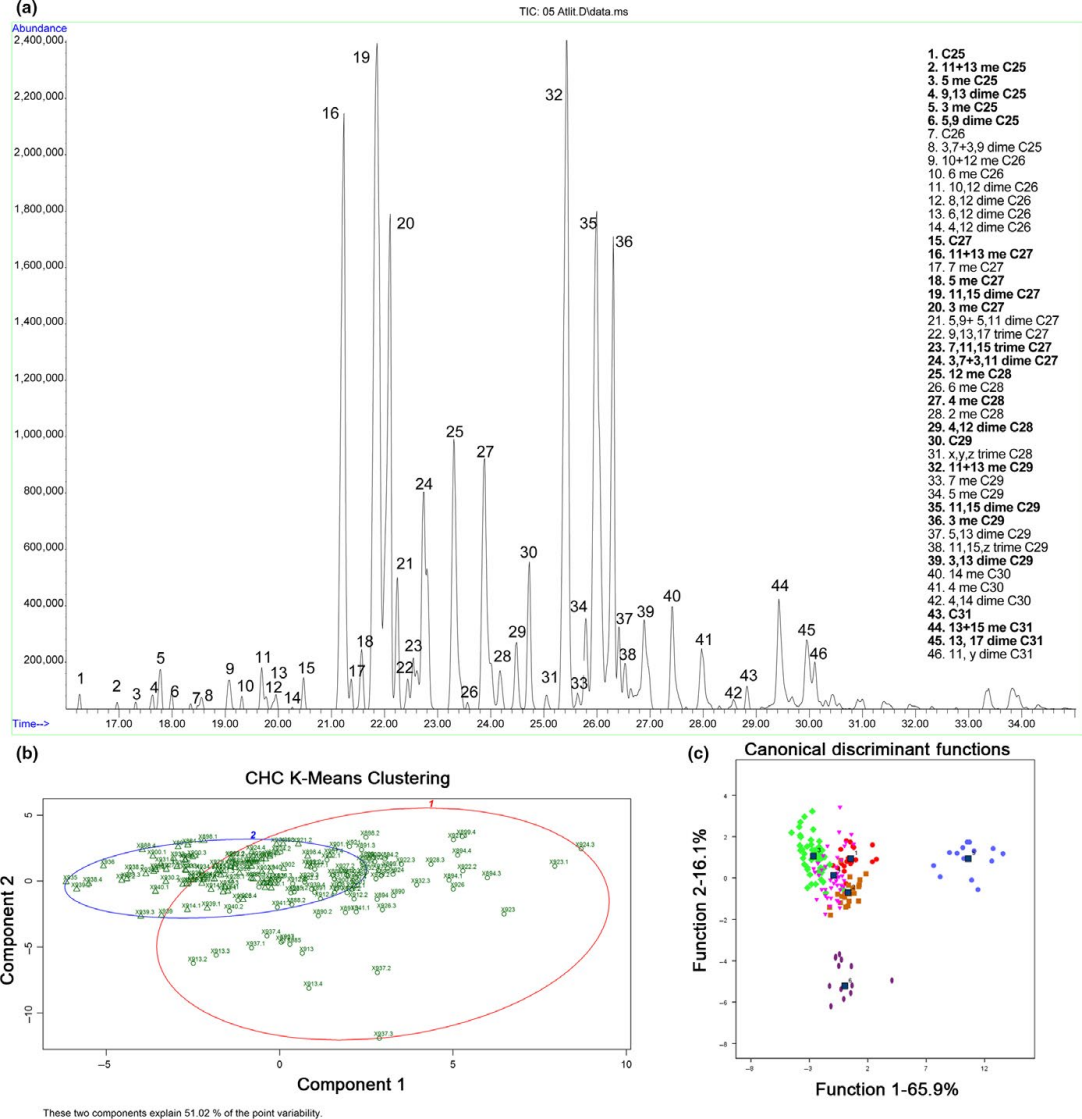
(a) Typical cuticular hydrocarbon profile of *C. israelensis*. The chromatogram shows only the 46 identified hydrocarbons (numbered peaks), of which 24 hydrocarbons were used in the analysis (marked in bold). (b) *K*‐means of CHC profiles *K* = 2, individuals are marked with nest number (first 3 digits) and individual ID. Circle indicates *k*‐1, triangle *k*‐2. The two components explain 51.02% of the variation. (c) Discriminant analysis based on CHC profiles while pooling nests to their assigned genetic colony (colored as depicted in Figure 1c), as indicated by STRUCTURE (*k* = 6)

Analyses of the hexane head extracts revealed a complex profile comprising 24 identified hydrocarbons (Figure 2a), accompanied by several branched fatty acids, alcohols, and esters (not shown in the chromatogram). Using the relative abundance of the different hydrocarbons, we first performed a principle component analysis that gave very little information, due to the small differences between samples (Supporting Information Figure S1). We then used the *k*‐means approach to determine the level of structuring in the samples. The most probable number of *K*'s was two according to all three analysis methods used, with the two components explaining only 51% of the variability (Figure 2b). Discriminant analysis was also performed while assigning the nests to colonies according to the results obtained by the STRUCTURE program. As depicted in Figure 2c, separation between colonies was significant (Wilks Lambada *p* < 0.05; function 1 explains 65.9% and function 2 16.1% of the variance, respectively). There was, however, also a clear distinction between sites. Colonies five and six, which were very distinct from all other colonies, originated from the Habonim population; while those from the Atlit population were clumped together, but still distinct from each other.

## 4 BEHAVIORAL ASSAYS

Nest merging was carried out between QR nests of differing proximities (8–100 m; Table 1). In the assays involving nests that were far apart (90 and 100 m, respectively) workers showed only mild mutual aggression, and even entered each other's nests without any obvious reaction by the resident workers. However, on the third day the respective queens of both nests were executed, and a few days later also 10 to 20 of the workers (from each nest) were killed, indicating that the nests belonged to different colonies. In nests that were spatially nearer to each other (15 meters or less) we observed workers entering each other's nests and noted mutual high aggression between workers and from workers to queens (10–20 worker‐worker attacks in 10 min); aggression was also noted in the common foraging arena. Because the queens were heavily attacked, we stopped the tests before they were injured. In the assays involving QR (nests 100, 101, and 102) and neighboring QL nests (nests 103, 104, and 105) all the QL nests merged only with QR nest 102, which was located 2, 5, and 11 meters away from them, respectively.

### 4.1 Association between genetic, chemical, and geographic distances

**Figure 3:**
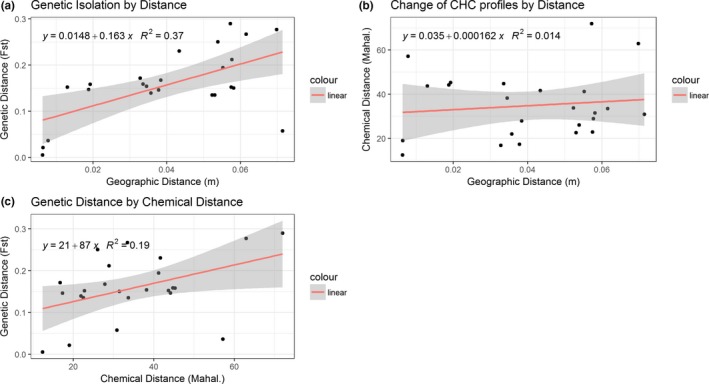
Regression analyses between cuticular hydrocarbon profiles (a) and *F*_st_ values (b), respectively, and metric distances between nests. Red line indicates linear regression, gray indicates 95% confidence interval (*R* ^2^ = 0.014 and 0.37 respectively). (c) Correlation between CHC and genetic distances

Figure 3a,b depicts the correlation between geographic and chemical or genetical distances, and Figure 3c the correlation between the chemical and genetical distances. There was no correlation between geographic and chemical distance (*R* ^2^ = 0.014; Pearson *p* value = 0.03; Figure 3a). In contrast, there was a positive correlation between the genetic *F*_st_'s and the geographic distance between the nests (*R* ^2^ = 0.37; Pearson *p* value ≈ 0 Figure 3b), and a slight positive correlation between the chemical and genetic distances (*R* ^2^ = 0.19; Pearson *p* value ≈ 0 Figure 3c). Mantel test gave comparable results: there was low correlation between chemical and geographical distances (*r* = 0.124, *p* = 0.115), and positive correlation between genetic with geographic distances (*r* = 0.427, p≈0), as well as between chemical and genetic distances (*r* = 0.39, p≈0).

Calculation of the nearest‐neighbor extrapolated‐nest‐density revealed an *R* value of 0.419 (*R* = mean distance of nearest neighbor observed/expected), indicating that the nests are aggregated more than expected under random distribution. We therefore checked for nest clusters using cluster analysis based on average geographic distances between nests (GPS locations). In Atlit there were eight main clusters of nests that were less than 20 meters apart from each other. Using the same nests, we performed a second cluster analysis based on average genetic distances (*F*_st_'s). In this clustering, we obtained a slightly larger number of clusters (12 main clusters; Supporting Information Figure S2), with *F*_st_ < 0.05. Aligning the two dendograms into a tanglegram resulted in a high level of entanglement (*E* = 0.63 in Atlit only), indicating a poor match between the dendograms. A third cluster analysis with the same nest population was performed using the average chemical Mahalanobis distances identified nine clusters (*x* < 15), but nest composition of each of these clusters differed from that based on geographic distances, as revealed by the high‐entanglement value (0.75). Finally, comparison of the dendograms based on genetic and chemical distances similarly resulted in a mismatch with an entanglement value of 0.64.

## 5 DISCUSSION

The newly‐described *Cataglyphis israelensis* is distributed along the central mountain ridge in Israel, but has also a small extension along the coast, at the foot of Mount Carmel, where it is sympatric with *C. savignyi*. In this study, we determined the social and population structure of *C. israelensis *in its coastal distribution. The results of nest excavations indicate that this species is monogynous. This was also supported by the genetic analysis of several nests where all workers’ genotypes matched that of their mother queen. However, in some of the nests, workers’ genotypes did not correspond with the assumed queen's genotypes. Two possible explanations can account for this genotype discrepancy. Some of the nest may have had multiple queens, which we may have missed during excavation. Alternatively, these workers may represent drifting workers that entered and were accepted in the wrong nest. The phenomenon of drifting workers is also known to occur in other *Cataglyphis* species (M. Knaden, personal communications), as well as other social insects (wasps, Sumner, Lucas, Barker, & Isaac, 2007; bumblebees, Lopez‐Vaamonde, Koning, Brown, Jordan, & Bourke, 2004; and bees, Bordier, Pioz, Crauser, Conte, & Alaux, 2017). In a viscose population with very similar CHC profiles as we observed in our case, it could be elevated due to higher chance for individual‐level mistakes. Drifting of workers may be also adaptive, being associated with mechanisms to increase within nest genetic diversity and a form of social parasitism (Nonacs, 2017; Zanette, Miller, Faria, Lopez‐Vaamonde, & Bourke, 2014). On top of that, the queens are polyandrous, mating with five males on average. This concurs with findings for most of the *Cataglyphis* species studied to date (Boulay et al., 2017). Nest abundance in the studied populations was 0.00185 nests/m^2^, with each nest comprising 200 to 2,000 workers. To unravel the social and population structure of *C. israelensis* at the study site, we employed three different methods. First, genetic microsatellite analysis (STRUCTURE) indicated a highly polydomous population, and possibly a supercolony. However, although the mean *F*_st_ was rather low (0.15), it was not as low as that of a previously described supercolony of *C. niger* (0.005; (Leniaud et al., 2011), but more similar to that described for the monodomous populations of *C. savignyi* and *C. livida* (0.14 and 0.21, respectively (Timmermans et al., 2010; Leniaud et al., 2011)). Moreover, these *F*_st_ values, along with our finding that they are positively correlated with distance between nests, can also be interpreted as a result of isolation by distance. We suggest that because this population is at the species’ distribution edge, as well as having fragmented distribution due to increasing land use for construction etc., or to different ecological niches, nests are somewhat isolated and gynes have difficulty dispersing. Alternatively, the genetic gradient with distance could be the consequence of their mating strategy, such as dispersal by budding, cloning of queens, or simply reduced flight ability of the gynes, as described for other *Cataglyphis* species. It follows that unraveling only the genetic structure of colonies provides a rather ambiguous estimate of population structure. There may be several explanations of why the STRUCTURE program collapsed nests into a unicolonial structure. Within‐nest relatedness was rather low (0.3) due to the high number of matings per queen, but the *F*_st_ values still differed from zero, indicating a genetic separation between nests. Thus, colonies can be closely related but nonetheless do not form a unicolonial structure. This might also be due to a reasonably high level of drifting workers. Comparing nests with similar matrilines (using COLONY) seems to give a more accurate and biologically meaningful result, however, because the method of STRUCTURE is commonly used, the comparison is noteworthy.

Similar results were obtained from the cuticular hydrocarbon analyses, with most of the nests, mainly from the Atlit population, demonstrating similar profiles, as expected from a unicolonial structure. Despite this profile similarity, the discriminant analysis was still able to discriminate several colonies. However, some of the nests that exhibited similar profiles were geographically remote from each other (and thus cannot be considered to share the same colony, unless they are part of a very large supercolony). CHC profiles are influenced both genetically and environmentally. Thus, in a small area like the one examined here, the possibility of isolation by distance (as suggested from the genetic data) superimposed by similarity in prey composition, may make differences between colonies hard to detect. Another variable to consider is that in our analysis we examined only the hydrocarbon constituents (following their demonstrated role in nest insularity in the closely related species *C. niger* (Lahav et al., 1999), but we cannot exclude the possibility that in this species polar compounds too are used for colonial identity.

The third method for delineating colony boundaries used in this study was to examine behavioral nest insularity/openness. Although, when given the opportunity, the presumed allocolonial ants entered each other's nests without demonstrating overt mutual aggression, they did not really merge into one large colony but, rather, invaded the opposite nest and killed its respective queen. Hence, these results refute any option of the existence of a supercolony structure, as may have otherwise been interpreted from the genetic or chemical analyses alone. The results of the experiments in which the queenless nests readily merged with a nearby queenright nest are consistent with a polydomous population structure.

To clarify some of the ambiguity in the results obtained by the different methods used in the study, we carried out a comparison among these methods. We first clustered the nests into assumed colonies according to either their physical location or microsatellite composition, or CHC profiles, and then determined whether these colony assignments were aligned. The underlying assumption for such a comparison is that the population structure is polydomous and that polydomous nests should be located in close proximity to each other. Neither the cluster based on microsatellites profiles nor that based on CHC profiles gave complete alignment with the cluster based on distances between nests. Furthermore, the finding that the microsatellite analysis did not match that of the CHC analysis indicates that CHC composition is not influenced by genetic composition alone. The comparisons further indicate that each method, when used individually for colony delineation, gives slightly different results. However, we cannot exclude the possibility that our markers may not have been sensitive enough to determine population structure of this small and somewhat isolated and viscous population.

In conclusion, the most probable population structure of *C. israelensis* along the coastline of Atlit is that of polydomy, with each colony consisting in one main nest, in which the single queen is located, and featuring several additional satellite nests, apparently queenless. This population structure seems to be very common in this genus and habitat, especially in the bicolor group (*C. savignyi*; Saar et al., 2014, *C. drusus; *unpublished data). The basis for such population structure could be due to difficulty of dispersal, properties of the substrate, and/or richness of the habitat. When isolation by distance occurs, new gynes hardly disperse and daughter colonies are located in high proximity to mother colonies. In soft substrate like sand, it is easier to dig new nests and, therefore, nest turnover may be large. Finally, a high availability of food increases nest mass and is perhaps conducive to colony spread.

Our study also emphasizes the importance of using a multi‐method approach in population structure studies. A multi‐method approach is often used for delimiting species’ boundaries and we suggest its use also in delimiting colony boundaries. Most of the methods frequently used to determine a specific colony signature are affected by factors such as isolation by distance, food abundance and distribution, social structure, and mating systems. Thus, using a single method for assessment is insufficient and the importance of behavioral assays cannot be overemphasized.

## AUTHORS’ CONTRIBUTIONS

TRB and AH conceived the ideas and designed the methodology; TRB collected the data; TRB analyzed the genetic and behavioral data; TRB and AH analyzed the chemical data; TRB and AH led the writing of the manuscript. All authors contributed critically to the drafts and gave final approval for publication.

## DATA ACCESSIBILITY

Sampling locations, CHC data and microsatellite genotypes are available at: Dryad https://doi.org/10.5061/dryad.6gb3977.

## References

[ece34535-bib-0001] Agosti, D. (1990). Review and reclassification of *Cataglyphis* (Hymenoptera, Formicidae). Journal of Natural History, 24, 1457–1505.

[ece34535-bib-0002] Amor, F. , Ortega, P. , Cerdá, X. , & Boulay, R. (2010). Cooperative prey‐retrieving in the ant *Cataglyphis floricola*: An unusual short‐distance recruitment. Insectes Sociaux, 57(1), 91–94. 10.1007/s00040-009-0053-x

[ece34535-bib-0003] Boomsma, J. J. (2009). Lifetime monogamy and the evolution of eusociality. Philosophical Transactions of the Royal Society of London B: Biological Sciences, 364, 3191–3207. 10.1098/rstb.2009.0101 19805427 PMC2781870

[ece34535-bib-0004] Bordier, C. , Pioz, M. , Crauser, D. , Le Conte, Y. , & Alaux, C. (2017). Should I stay or should I go: Honeybee drifting behaviour as a function of parasitism. Apidologie, 48, 286–297. 10.1007/s13592-016-0475-1.

[ece34535-bib-0005] Boulay, R. , Aron, S. , Cerdá, X. , Doums, C. , Graham, P. , Hefetz, A. , & Monnin, T. (2017). Social life in arid environments: The case study of *Cataglyphis* ants. Annual Review of Entomology, 62, 305–321. 10.1146/annurev-ento-031616-034941 27860520

[ece34535-bib-0006] Boulay, R. , Carro, F. , Soriguer, R. C. , & Cerdá, X. (2007). Synchrony between fruit maturation and effective dispersers’ foraging activity increases seed protection against seed predators. Proceedings of the Royal Society B: Biological Sciences, 274, 2515–2522. 10.1098/rspb.2007.0594. PMC2275878 17698486

[ece34535-bib-0007] Boulay, R. , Katzav‐Gozansky, T. , Vander Meer, R. K. , & Hefetz, A. (2003). Colony insularity through queen control on worker social motivation in ants. Proceedings of the Royal Society B: Biological Sciences, 270, 971–977. 10.1098/rspb.2002.2325 PMC1691331 12803913

[ece34535-bib-0008] Bourke, A. F. , & Franks, N. R. (1995). Social evolution in ants. Princeton, NJ: Princeton University Press.

[ece34535-bib-0009] Buczkowski, G. , Kumar, R. , Suib, S. L. , & Silverman, J. (2005). Diet‐related modification of cuticular hydrocarbon profiles of the argentine ant, *Linepithema humile*, diminishes intercolony aggression. Journal of Chemical Ecology, 31, 829–843. 10.1007/s10886-005-3547-7 16124254

[ece34535-bib-0010] Cerda, X. , Retana, J. , & Cros, S. (1997). Thermal disruption of transitive hierarchies in Mediterranean ant communities. Journal of Animal Ecology, 363–374.

[ece34535-bib-0011] Clark, P. J. , & Evans, F. C. (1954). Distance to nearest neighbor as a measure of spatial relationships in populations. Ecology, 35, 445–453. 10.2307/1931034

[ece34535-bib-0012] Clémencet, J. , & Doums, C. (2007). Habitat‐related microgeographic variation of worker size and colony size in the ant *Cataglyphis cursor* . Oecologia, 152, 211–218. 10.1007/s00442-006-0646-2. 17245588

[ece34535-bib-0013] Crozier, R. H. , & Pamilo, P. (1996). Evolution of social insect colonies. Oxford, UK: Oxford University Press.

[ece34535-bib-0014] Darras, H. , Leniaud, L. , & Aron, S. (2014). Large‐scale distribution of hybridogenetic lineages in a Spanish desert ant. Proceedings of the Royal Society of London B: Biological Sciences 281, 20132396. 10.1098/rspb.2013.2396 PMC3843834 24225458

[ece34535-bib-0015] Earl, D. A. (2012). STRUCTURE HARVESTER: A website and program for visualizing STRUCTURE output and implementing the Evanno method. Conservation Genetics Resources, 4, 359–361.

[ece34535-bib-0016] Edgar, R. C. (2004). MUSCLE: Multiple sequence alignment with high accuracy and high throughput. Nucleic Acids Research, 32(5), 1792–1797. 15034147 10.1093/nar/gkh340 PMC390337

[ece34535-bib-0017] Ellis, S. , Procter, D. , Buckham‐Bonnett, P. , & Robinson, E. (2017). Inferring polydomy: A review of functional, spatial and genetic methods for identifying colony boundaries. Insectes Sociaux, 64, 19–37. 10.1007/s00040-016-0534-7 28255180 PMC5310590

[ece34535-bib-0018] Eyer, P.‐A. , Leniaud, L. , Darras, H. , & Aron, S. (2013). Hybridogenesis through thelytokous parthenogenesis in two *Cataglyphis* desert ants. Molecular Ecology, 22, 947–955. 23216892 10.1111/mec.12141

[ece34535-bib-0019] Eyer, P. A. , Seltzer, R. , Reiner‐Brodetzki, T. , & Hefetz, A. (2017). An integrative approach to untangling species delimitation in the *Cataglyphis bicolor* desert ant complex in Israel. Molecular Phylogenetics and Evolution, 115, 128–139. 10.1016/j.ympev.2017.07.024 28774791

[ece34535-bib-0020] Goudet, J. (1995). FSTAT (version 1.2): A computer program to calculate F‐statistics. Journal of Heredity, 86, 485–486.

[ece34535-bib-0021] Hamilton, W. D. (1964). The genetical evolution of social behaviour. II. Journal of Theoretical Biology, 7, 17–52. 5875340 10.1016/0022-5193(64)90039-6

[ece34535-bib-0022] Hamilton, W. D. (1972). Altruism and related phenomena mainly in the social insects. Annual Review of Ecology and Systematics, 2, 193–232.

[ece34535-bib-0023] Hefetz, A. (2007). The evolution of hydrocarbon pheromone parsimony in ants (Hymenoptera: Formicidae)—interplay of colony odor uniformity and odor idiosyncrasy. Myrmecological News, 10, 59–68.

[ece34535-bib-0024] Heinze, J. (2008). The demise of the standard ant (Hymenoptera: Formicidae). Myrmecological News, 11, 9–20.

[ece34535-bib-0025] Hölldobler, B. , & Wilson, E. O. (1990). The ants. Cambridge, MA: Harvard University Press.

[ece34535-bib-0026] Ionescu, A. , & Eyer, P. (2016). Notes on *Cataglyphis Foerster*, 1850 of the bicolor species‐group in Israel, with description of a new species (Hymenoptera: Formicidae). Israel Journal of Entomology, 46, 109–131.

[ece34535-bib-0027] Jones, O. R. , & Wang, J. (2010). COLONY: A program for parentage and sibship inference from multilocus genotype data. Molecular Ecology Resources, 10, 551–555. 10.1111/j.1755-0998.2009.02787.x 21565056

[ece34535-bib-0028] Jowers, M. J. , Leniaud, L. , Cerdá, X. , Alasaad, S. , Caut, S. , Amor, F. , … Boulay, R. R. (2013). Social and population structure in the ant *Cataglyphis emmae* . PloS One, 8, e72941 10.1371/journal.pone.0072941 24039827 PMC3767659

[ece34535-bib-0029] Keller L. (Ed.) (1993). Queen number and sociality in insects (pp. 16–44). Oxford: Oxford University Press.

[ece34535-bib-0030] Kennedy, P. , Baron, G. , Qiu, B. , Freitak, D. , Helanterä, H. , Hunt, E. R. , … Sumner, S. (2017). Deconstructing superorganisms and societies to address big questions in biology. Trends in Ecology & Evolution, 32, 861–872. 10.1016/j.tree.2017.08.004 28899581

[ece34535-bib-0031] Lahav, S. , Soroker, V. , Hefetz, A. , & Vander Meer, R. K. (1999). Direct behavioral evidence for hydrocarbons as ant recognition discriminators. Naturwissenschaften, 86, 246–249.

[ece34535-bib-0032] Leniaud, L. , Darras, H. , Boulay, R. , & Aron, S. (2012). Social hybridogenesis in the clonal ant *Cataglyphis hispanica* . Current Biology, 22, 1188–1193. 10.1016/j.cub.2012.04.060 22683263

[ece34535-bib-0033] Leniaud, L. , Heftez, A. , Grumiau, L. , & Aron, S. (2011). Multiple mating and supercoloniality in *Cataglyphis* desert ants. Biological Journal of the Linnean Society, 104, 866–876. 10.1111/j.1095-8312.2011.01772.x

[ece34535-bib-0034] Lenoir, A. , Aron, S. , Cerda, X. , & Hefetz, A. (2009). *Cataglyphis* desert ants: A good model for evolutionary biology in Darwin’s anniversary year–A review. Israel Journal of Entomology, 39, 1–32.

[ece34535-bib-0035] Liang, D. , & Silverman, J. (2000). “You are what you eat”: Diet modifies cuticular hydrocarbons and nestmate recognition in the Argentine ant, *Linepithema humile* . Naturwissenschaften, 87, 412–416. 10.1007/s001140050752 11091966

[ece34535-bib-0036] Lopez‐Vaamonde, C. , Koning, J. W. , Brown, R. M. , Jordan, W. C. , & Bourke, A. F. G. (2004). Social parasitism by male‐producing reproductive workers in a eusocial insect. Nature, 430, 557–560. 10.1038/nature02769. 15282605

[ece34535-bib-0037] Nonacs, P. (2017). Go high or go low? Adaptive evolution of high and low relatedness societies in social hymenoptera. Frontiers in Ecology and Evolution, 5, 10.3389/fevo.2017.00087

[ece34535-bib-0038] Pearcy, M. , Clémencet, J. , Chameron, S. , Aron, S. , & Doums, C. (2004). Characterization of nuclear DNA microsatellite markers in the ant *Cataglyphis cursor* . Molecular Ecology Resources, 4, 642–644. 10.1111/j.1471-8286.2004.00759.x

[ece34535-bib-0039] Pritchard, J. K. , Stephens, M. , & Donnelly, P. (2000). Inference of population structure using multilocus genotype data. Genetics, 155, 945–959. 10835412 10.1093/genetics/155.2.945 PMC1461096

[ece34535-bib-0040] Procter, D. S. , Cottrell, J. E. , Watts, K. , A'Hara, S. W. , Hofreiter, M. , & Robinson, E. J. (2016). Does cooperation mean kinship between spatially discrete ant nests? Ecology and Evolution, 6, 8846–8856. 10.1002/ece3.2590 28035273 PMC5192893

[ece34535-bib-0041] Queller, D. C. (1992). A general model for kin selection. Evolution, 46, 376–380. 10.1111/j.1558-5646.1992.tb02045.x 28564031

[ece34535-bib-0042] Raymond, M. , & Rousset, F. (1995). *GENEPOP on the Web (Version 3.4)* . Retrieved from http//wbiomed.curtin.edu.au/genepop/.

[ece34535-bib-0043] Ryan, S. F. , Fontaine, M. C. , Scriber, M. J. , Pfrender, M. E. , O'Neil, S. T. , & Hellmann, J. J. (2017). Patterns of divergence across the geographic and genomic landscape of a butterfly hybrid zone associated with a climatic gradient. Molecular Ecology, 26(18), 4725–4742. 28727195 10.1111/mec.14236

[ece34535-bib-0044] Saar, M. , Leniaud, L. , Aron, S. , & Hefetz, A. (2014). At the brink of supercoloniality: Genetic, behavioral, and chemical assessments of population structure of the desert ant *Cataglyphis niger* . Frontiers in Ecology and Evolution, 2, 13.

[ece34535-bib-0045] Simon, C. , Frati, F. , Beckenbach, A. , Crespi, B. , Liu, H. , & Flook, P. (1994). Evolution, weighting, and phylogenetic utility of mitochondrial gene sequences and a compilation of conserved polymerase chain reaction primers. Annals of the Entomological Society of America, 87(6), 651–701. 10.1093/aesa/87.6.651

[ece34535-bib-0046] Soroker, V. , Vienne, C. , Hefetz, A. , & Nowbahari, E. (1994). The postpharyngeal gland as a “Gestalt” organ for nestmate recognition in the ant *Cataglyphis niger* . Naturwissenschaften, 81, 510–513. 10.1007/s001140050120

[ece34535-bib-0047] Sumner, S. , Lucas, E. , Barker, J. , & Isaac, N. (2007). Radio‐tagging technology reveals extreme nest‐drifting behavior in a eusocial insect. Current Biology, 17, 140–145. 10.1016/j.cub.2006.11.064 17240339

[ece34535-bib-0048] Timmermans, I. , Grumiau, L. , Hefetz, A. , & Aron, S. (2010). Mating system and population structure in the desert ant *Cataglyphis livida* . Insectes Sociaux, 57, 39–46. 10.1007/s00040-009-0048-7

[ece34535-bib-0049] Timmermans, I. , Hefetz, A. , Fournier, D. , & Aron, S. (2008). Population genetic structure, worker reproduction and thelytokous parthenogenesis in the desert ant *Cataglyphis sabulosa* . Heredity, 101, 490. 19018270 10.1038/hdy.2008.72

[ece34535-bib-0050] Vonshak, M. , Dayan, T. , Foucaud, J. , Estoup, A. , & Hefetz, A. (2009). The interplay between genetic and environmental effects on colony insularity in the clonal invasive little fire ant *Wasmannia auropunctata* . Behavioral Ecology and Sociobiology, 63, 1667–1677. 10.1007/s00265-009-0775-9

[ece34535-bib-0051] Wang, J. (2011). COANCESTRY: A program for simulating, estimating and analysing relatedness and inbreeding coefficients. Molecular Ecology Resources, 11, 141–145. 21429111 10.1111/j.1755-0998.2010.02885.x

[ece34535-bib-0052] Wehner, R. (2008). The desert ant's navigational toolkit: Procedural rather than positional knowledge. Navigation, 55(2), 101–114. 10.1002/j.2161-4296.2008.tb00421.x

[ece34535-bib-0053] Wilson, E. O. (2012). The social conquest of earth. New York, NY: WW Norton & Company.

[ece34535-bib-0054] Wright, S. (1943). Isolation by distance. Genetics, 28, 114. 17247074 10.1093/genetics/28.2.114 PMC1209196

[ece34535-bib-0055] Zanette, L. R. S. , Miller, S. D. L. , Faria, C. M. A. , Lopez‐Vaamonde, C. , & Bourke, A. F. G. (2014). Bumble bee workers drift to conspecific nests at field scales. Ecological Entomology, 39, 347–354. 10.1111/een.12109.

