# Supplementary figures and images for: Determining social and population structures requires multiple approaches: A case study of the desert ant Cataglyphis israelensis
Source: Ecol Evol. 2018 Dec 10;8(24):12365–74. doi: 10.1002/ece3.4535 (PMC6308896; doi:10.1002/ece3.4535)

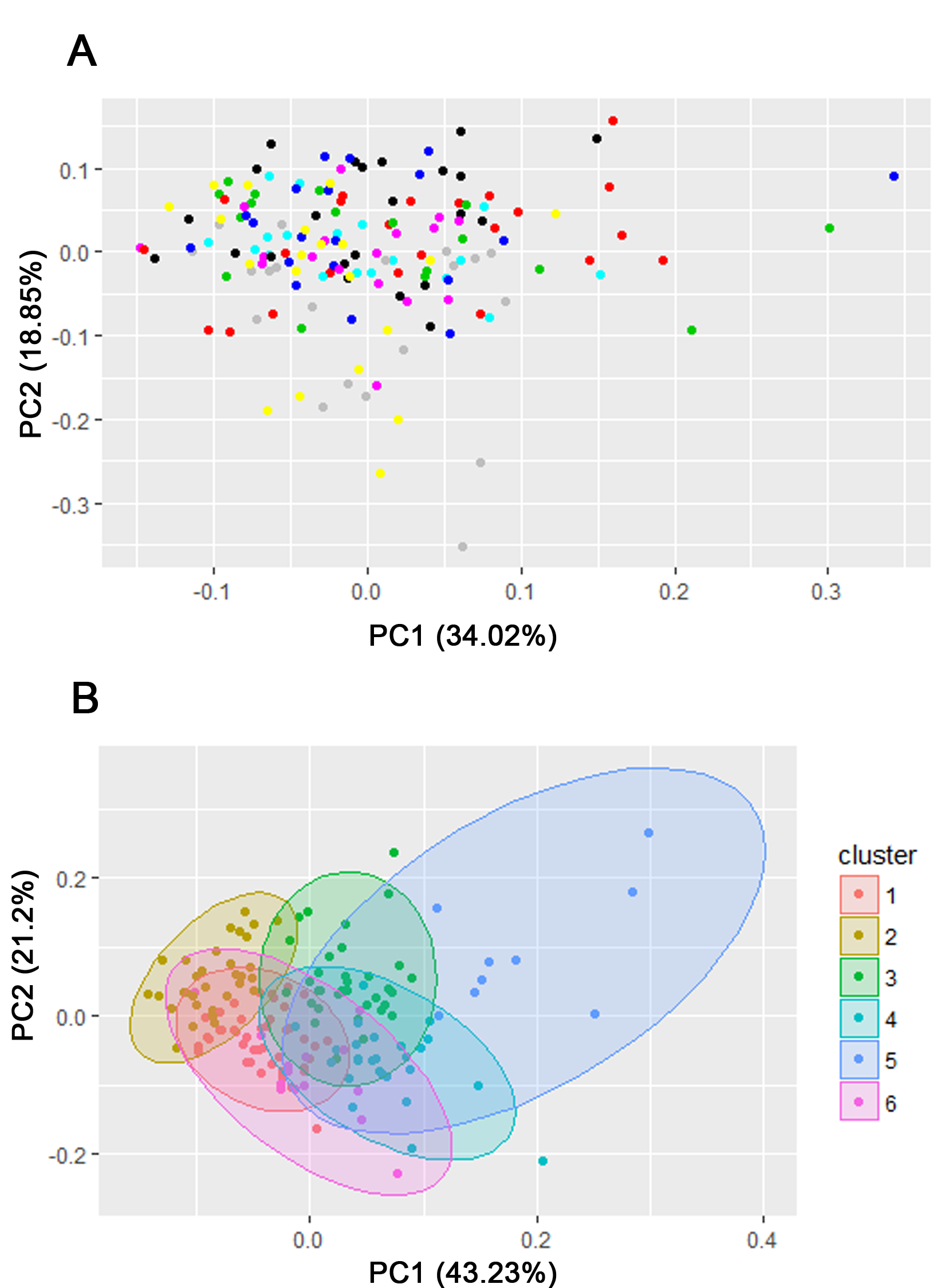

Supplement: Supplementary file 1 [file ECE3-8-12365-s001.tif]

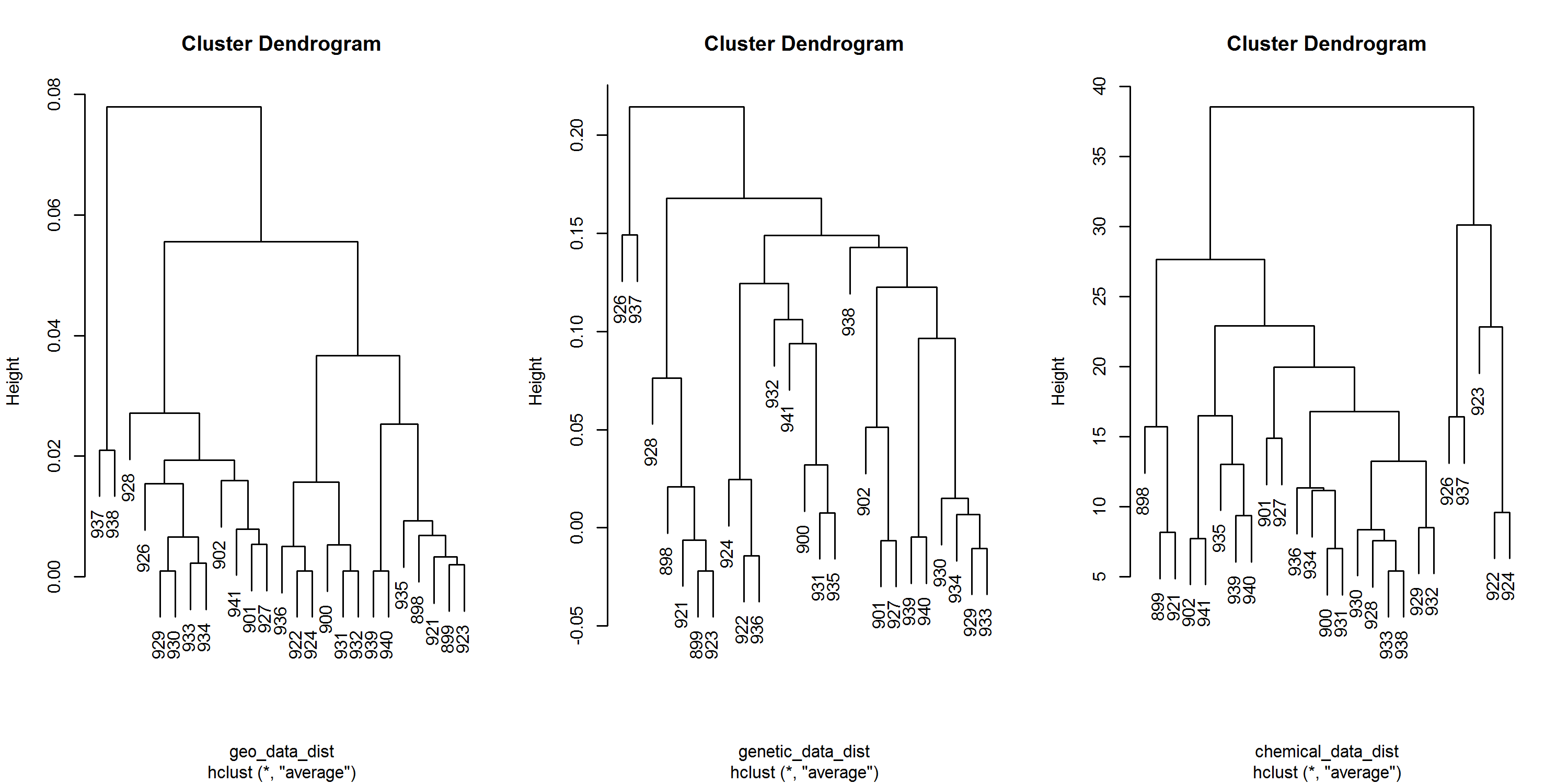

Supplement: Supplementary file 2 [file ECE3-8-12365-s002.tiff]

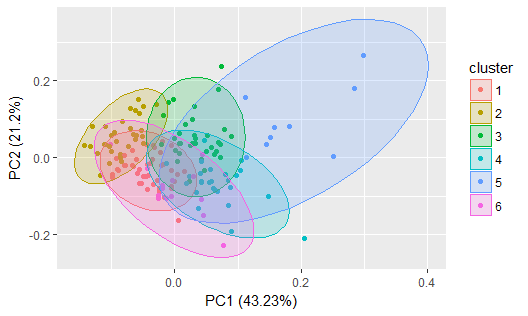

Supplement: Supplementary file 3 [file ECE3-8-12365-s003.tiff]
