# Supplementary material for: Determining social and population structures requires multiple approaches: A case study of the desert ant Cataglyphis israelensis
Source: Ecol Evol. 2018 Dec 10;8(24):12365–74. doi: 10.1002/ece3.4535 (PMC6308896; doi:10.1002/ece3.4535)
Supplement: Supplementary file 4 [file ECE3-8-12365-s004.docx]

Table S1: Number of nests used in each of the analyses according to location of collection site.

| Table ST1 |  |  |  |  |  |
| --- | --- | --- | --- | --- | --- |
|  |  |  |  |  |  |

Table S2: Reconstructed matriline multilocus genotypes in Atlit as suspected by COLONY software
